# Utility of Gastric Lavage in Vigorous Neonates Delivered with Meconium Stained Liquor: A Randomized Controlled Trial

**DOI:** 10.1155/2014/204807

**Published:** 2014-04-24

**Authors:** Jatin Garg, Rupesh Masand, Balvir Singh Tomar

**Affiliations:** Department of Pediatrics, National Institute of Medical Sciences, 4 Govind Marg, NIMS City Center, Jaipur, Rajasthan 302004, India

## Abstract

*Objective*. To determine the incidence of feed intolerance in vigorous babies with meconium stained liquor (MSL) who received prophylactic gastric lavage as compared to those who were not subjected to this procedure. *Design*. Randomized controlled trial. *Setting*. Tertiary care teaching hospital. *Participants/Intervention*. 330 vigorous babies delivered with MSL and satisfying the predefined inclusion criteria were randomized either to receive gastric lavage (group A, *n* = 165) or to not receive gastric lavage (group B, *n* = 153). Clinical monitoring was subsequently performed and recorded in prestructured proforma. *Results*. There was no significant statistical difference (*P* > 0.05) in incidence of feed intolerance in “lavage” and “no lavage” groups. *Secondary Outcome*. There was no evidence of secondary respiratory distress in either group. None of the patients in the lavage group exhibited adverse effects owing to the procedure. *Conclusions*. There is no role of prophylactic gastric lavage in neonates born with MSL.

## 1. Introduction

Meconium is a blackish-green sticky material composed of debris of intestinal cells, lanugo hair, vernix, liquor, and bile pigments [1, 2]. The incidence of meconium stained liquor (MSL) varies between 7% and 22% of life births [3–7]. Although unsubstantiated, it is thought that the presence of meconium in the stomach can act as a chemical irritant and can cause feeding problems [8]. These are 2.8 times more frequent in neonates born with MSL than those born without it, regardless of consistency of the amniotic fluid [9]. It has been hypothesized that some cases of meconium aspiration syndrome might be caused by postnatal aspiration of gastric contents into the airways [10]. Gastric lavage has been routinely employed with this belief to evacuate the gastric contents and avoid feeding problems but like other procedures it has been associated with complications [8, 11, 12].

Looking at the almost universal practice [11] of prophylactic gastric lavage in neonates delivered with MSL and its recommendation by pediatric textbooks [12–15], despite negligible scientific evidence and evidence-based recommendations, this study was designed with the objective of determining if gastric lavage in well babies with MSL led to the development of less feed intolerance as compared to those who were not subjected to this procedure.

## 2. Methodology

This randomized control trial was conducted in NICU of a tertiary care teaching hospital between August 2011 and July 2012, after approval from The Institutional Ethical Committee. For the purpose of the study, 330 vigorous neonates delivered with MSL and bearing a birth weight ≥1800 grams and gestational age ≥34 weeks were included. Neonates with hypoxic ischemic encephalopathy (HIE), major congenital malformation, Downes' score [16] for respiratory distress >3, and requiring CPR at the time of birth were excluded. An informed written consent was obtained by the attending resident doctor from the precounseled parents/guardians, immediately after birth of their neonate, who satisfied the required study criteria.

**Figure 1:**
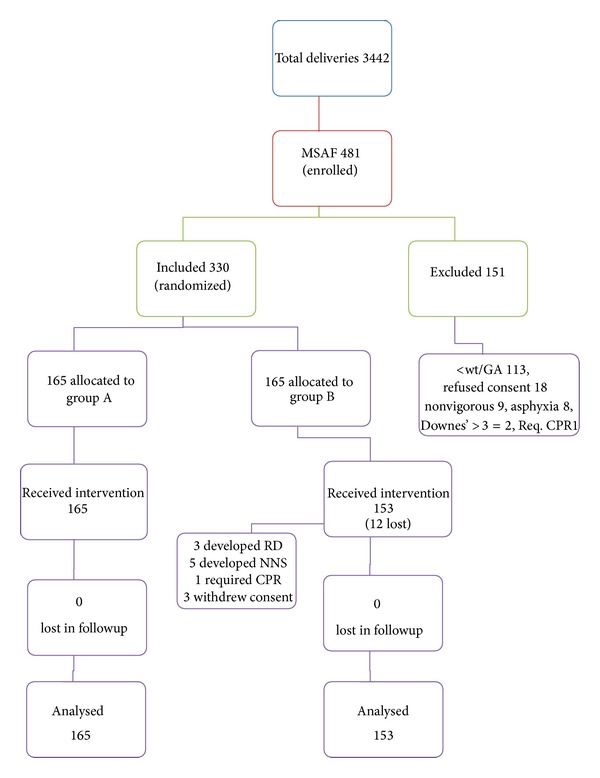
Study flow chart. MSL: meconium stained liquor; group A: the “gastric lavage” group; group B: the “no gastric lavage” group; GA: gestational age; <wt/GA: birth weight ≦1800 grams and gestational age ≦34 weeks; req. CPR: requiring cardiopulmonary resuscitation; RD: respiratory distress; NNS: neonatal sepsis.

After initial care and stabilization, neonates were randomized either to receive gastric lavage (group A) or to not receive lavage (group B). Randomization was done using small square slips with computer generated numbers from 1 to 330. These prenumbered slips were folded and shuffled in a box and opened for each neonate to decide the intervention. Neonates with odd-numbered slips were allotted group A, while withdrawal of even-numbered slips rendered the study subjects in group B. A sample size of 165 in each group with an *α* error of 5% and 90% power in a two sided test was required. Blinding of intervention/outcome was not done; that is, the doctors and nursery staff were aware of the intervention (Figure 1).

Details of name, age, sex, weight, gestational age, mode of delivery, and vital parameters pre- and postgastric lavage were recorded in a prestructured proforma by resident duty doctors.

In group A, all neonates were subjected to lavage using a nasogastric tube of 6Fr/8Fr size and 10 mL/kg normal saline with aliquots of 5 mL each time, till the fluid aspirated was grossly clear. One of the authors (Jatin Garg) conducted the procedure in the first 30 minutes after birth and it was recorded by the posted residents in the NICU on study proforma. Neonates in group B were not subjected to lavage. All babies were exclusively breast-fed on demand (group B) and after 1 hr of lavage under supervision of nursing staff who counseled the mothers and observed feed intolerance, if there was any. Feed intolerance was defined as (i) >2 vomiting episodes in 4 hr period or >3 in 24 hr and/or (ii) presence of abdominal distension defined as an increase in abdominal girth by 2 cm from baseline (checked only if repeated vomiting episodes were present) and/or (iii) if gastric residual volume is >2 mL of undigested milk or bile colored (checked only if abdominal distension noted) [17]. Neonates were monitored clinically for at least 15 minutes after the procedure and then subsequently at 1, 13, 24, and 48 hrs of life in the observation area of NICU. On every occasion, heart rate, respiratory rate, abdominal girth, gastric residue, vomiting episodes, chest examination, and signs of respiratory distress were noted and Downes' scoring was performed, if required. Neonates in lavage group were monitored for complications secondary to nasogastric tube insertion, like apnea, bradycardia, and trauma to the nasal cavity. Appropriate statistical analysis was performed using SPSS 17 software. *P* value <0.05 was considered significant.

## 3. Results

There was no significant statistical difference (*P* > 0.05) in the incidence of feed intolerance in group A (9.70%) and group B (13.73%) (Table 1). None of the baseline characteristics like sex, birth weight, gestational age, mode of delivery, and consistency of meconium were significantly associated (*P* > 0.05) with occurrence of feed intolerance in our study subjects with meconium stained liquor. There was no evidence of secondary respiratory distress in either group A or group B. None of the patients in the lavage group exhibited adverse effects owing to the procedure, that is, apnea, bradycardia, or any trauma to nasal or oral cavity.

## 4. Discussion

The performance of gastric lavage in neonates remains a common practice in India and has been mentioned in neonatal protocols of other regions of the world [18]. It is based on this belief that meconium acts as a chemical irritant in the stomach which can cause gastritis and secondary meconium aspiration syndrome upon regurgitation of gastric contents. Thus, gastric lavage was justified in order to prevent feed intolerance and to increase the success of breast-feeding during the first few hours of life. This randomized controlled trial, however, demonstrated that there was no significant difference in incidence of feed intolerance in the “gastric lavage” (group A) or the “no gastric lavage” (group B) group. The incidence of feed intolerance was 9.7% in the Group A as compared to 13.72% in the Group B (*P* > 0.05) which was comparable with other studies [17, 19]. This statistically insignificant difference in our study can be explained by the hypothesis proposed by Sharma et al. [19] that vigorous neonates have reduced exposure to meconium in-utero as compared to non-vigorous babies. Early feeding postnatally, further dilutes the meconium and its irritant properties. In our study, there was no association of feed intolerance to sex of the study subjects in either of the two groups as observed similarly by Cuello-García et al. [11]. Wiswell et al. [3] documented male neonates to be more prone to feed intolerance than female neonates (*P* = 0.022). There was no association of birth weight and gestational age with feed intolerance in either group which was similar to the observations by Ameta et al. [17].

Feed intolerance had no association with the consistency of the meconium, which was in consonance with other studies [11, 17].

None of the babies in the “no gastric lavage” (group B) developed secondary respiratory distress owing to pulmonary aspiration of meconium containing regurgitated gastric fluid which was similar to the observation by other studies [8, 17, 19]. This is contrary to the belief that neonates with MSL are prone to such complications if lavage is not carried out. Narchi and Kulaylat [8] concluded that neonates with MSL are not prone to develop secondary respiratory distress whether lavage is done or not done.

In the present study, gastric lavage was well tolerated in all subjects; that is, there were no procedural complications like apnea, bradycardia, or trauma to the nasal cavity. This finding was in consonance with other studies [8, 11, 17, 19]. However, Widstrom et al. [20] reported small elevation in mean arterial blood pressure, increased retching, and disrupted sequence of prefeeding behavior in neonates who had undergone gastric suction. The physiological side effects induced by gastric suction are minor, but it seemed to be unpleasant for the neonates [20], which could not be evaluated in this study. It has been demonstrated that the aspiration of the gastric contents through a catheter in newborns can be a noxious stimulus. All noxious stimuli especially if repeated can increase functional disorders in adulthood [21]. Inability to perform blinding and, on the part of the nursing staff, to differentiate between regurgitation and vomiting (in spite of prior training) constituted the shortcomings of this study.

## 5. Conclusion

This study is a randomized control trial which evaluated a common practice in neonatal care without availability of scientific evidence.

Gastric lavage has been mentioned as part of essential newborn care during management of babies with meconium aspiration [12–15]. However, this study demonstrated that feeding problems are not significant in neonates born with meconium stained liquor (MSL) and that there is no role of routine prophylactic gastric lavage in reducing their incidence. In resource poor settings, this may help in saving equipments, nursing time, clinical attention, and preventing procedure-related complications.

This study concludes that gastric lavage should be reserved for treating the rather rare occurrence of feed intolerance in neonates born with MSL instead of being performed on a routine prophylactic basis.

## Figures and Tables

**Table 1:** Comparison of baseline variables in study groups and their association with feed intolerance.

Total		Group A (165)	Group B (153)	*P*	Odd ratio	95% CI
Over all feed intolerance		**16**	**21**	**0.253**	**0.67**	**0.338–1.3475**
GA*	34–36 wks 6 days	Group A (98)	Group B (69)	*P*	Odd ratio	95% CI
		**9**	**12**	**0.1207**	**0.4803**	**0.1903**–**1.2126**
	37–40 wks	Group A (67)	Group B (84)	*P*	Odd ratio	95% CI
		**7**	**9**	**0.9578**	**0.9722**	**0.3421**–**2.7629**

B.Wt	<2 kg	Group A (21)	Group B (19)	*P*	Odd ratio	95% CI
		**7**	**5**	**0.6292**	**1.4000**	**0.3572**–**5.4874**
	2-3 kg	Group A (140)	Group B (122)	*P*	Odd ratio	95% CI
		**7**	**6**	**0.9878**	**1.0088**	**0.3296**–**3.0875**
	>3 kg	Group A (4)	Group B (12)	*P*	Odd ratio	95% CI
		**2**	**10**	**0.2032**	**0.200**	**0.0168**–**2.3864**

Gender	M	Group A (86)	Group B (80)	*P*	Odd ratio	95% CI
		**9**	**11**	**0.5171**	**0.7332**	**0.2867**–**1.8750**
	F	Group A (79)	Group B (73)	*P*	Odd ratio	95% CI
		**7**	**10**	**0.5574**	**0.7350**	**0.2628**–**2.0558**

MOD^#^	Vaginal	Group A (92)	Group B (93)	*P*	Odd ratio	95% CI
		**7**	**11**	**0.33**	**0.61**	**0.22**–**1.66**
	C/S	Group A (73)	Group B (60)	*P*	Odd ratio	95% CI
		**9**	**10**	**0.47**	**0.70**	**0.26**–**1.86**

COM**	Thick	Group A (40)	Group B (42)	*P*	Odd ratio	95% CI
		**7**	**8**	**0.85**	**0.90**	**0.29**–**2.76**
	Thin	Group A (125)	Group B (111)	*P*	Odd ratio	95% CI
		**9**	**13**	**0.23**	**0.58**	**0.23**–**1.42**

Group A: the “gastric lavage” group; group B: the “no gastric lavage” group; CI: confidence interval; GA*: gestational age; B.Wt: birth weight; wks: weeks; kg: kilograms; M: male; F: female; MOD^#^: mode of delivery; C/S: cesarean section; COM**: consistency of meconium.
